# Unexpected posterior tilt in extravascular implantable cardioverter-defibrillator leads: Lessons learned from 3 cases

**DOI:** 10.1016/j.hrcr.2025.01.010

**Published:** 2025-01-30

**Authors:** Jarkko Karvonen, Aino-Maija Vuorinen, Fabian Noti, Andreas Haeberlin, Alexander Breitenstein

**Affiliations:** 1 Heart and Lung Center, Helsinki University Hospital and University of Helsinki, Helsinki, Finland; 2 Department of Radiology, HUS Diagnostic Center, Helsinki University Hospital and University of Helsinki, Helsinki, Finland; 3 Department of Cardiology, Inselspital, Bern University Hospital and University of Bern, Bern, Switzerland; 4 Department of Cardiology, University Heart Center Zurich, Zurich, Switzerland

**Keywords:** Implantable cardioverter-defibrillator, Extravascular implantable cardioverter-defibrillator, Cardiac implantable electronic device, Defibrillation, Substernal, Cardiac imaging

**Key Teaching Points**

• Posterior tilting of the extravascular implantable cardioverter-defibrillator (EV-ICD) lead can occur post implantation without affecting device short-term performance.
• Despite tilting, the electrical parameters of EV-ICDs remained stable and no corrective measures were necessary in our cases.
• In cases of postoperative posterior tilting of an EV-ICD lead, computed tomography imaging offers more precise lead localization.
• A computed tomography scan may not be essential if echocardiography and chest radiography confirm no complications and device electrical parameters remain stable.

## Introduction

The extravascular implantable cardioverter-defibrillator (EV-ICD), a novel nontransvenous alternative to conventional ICD systems, was recently validated for its safety and efficacy in terminating ventricular arrhythmias.^1^^,^^2^ The high-voltage lead is placed substernally, allowing relatively low pacing and defibrillation thresholds. Consequently, the EV-ICD offers antitachycardia pacing and short-term pause-prevention pacing alongside defibrillation capabilities.

**Figure 1:**
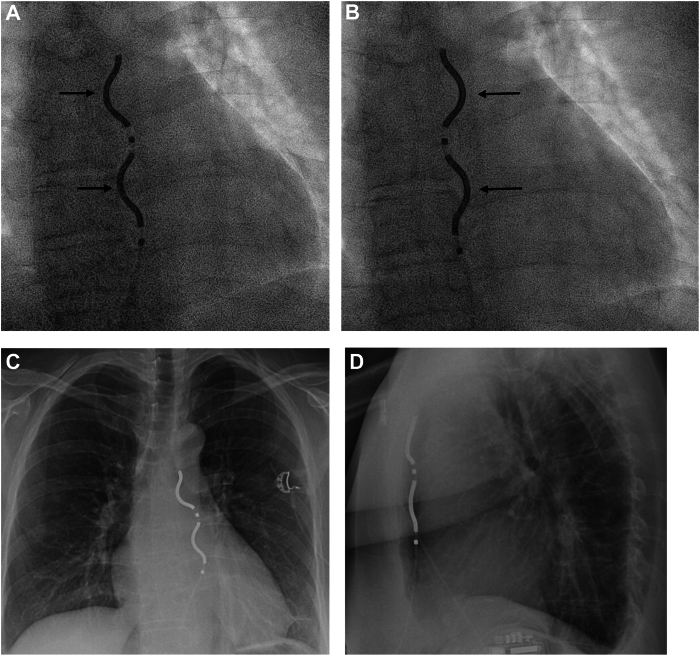
An example of flipping extravascular implantable cardioverter-defibrillator lead. **A:** Initial lead position immediately after introducer removal, with coils oriented to the right (*arrows*). **B:** The lead flipped 180 degrees within minutes, now with coils facing the left (*arrows*). The lead was removed and retunneled, aiming slightly more medially. **C, D:** Chest radiograph from the following day shows the final lead position near the sternum, with coils correctly oriented to the right. Note: These images are not associated with the reported cases.

The EV-ICD has an epsilon-shaped passive fixation lead,^3^ leaving the tip of the lead loose in the substernal space. The lead is implanted using a minimally invasive subxiphoid approach with a tunneling rod and a sheath under fluoroscopic guidance. The body of the lead is fixated by suturing an anchoring sleeve to the rectus fascia. Special care must be taken to ensure the correct orientation of the lead. This includes closely monitoring the movement of the lead tip after sheath removal to avoid inadvertent placement into the pericardial or pleural space. In addition, correct orientation of the lead is checked in the anteroposterior view, ensuring that the coils face toward the patient's right. It is recognized that the lead may unintentionally flip by 180 degrees after removing the sheath, resulting in coil orientation toward the left, necessitating a corrective action (Figure 1). This issue is addressed during implanter training.

We present a previously unreported observation encountered in 3 distinct cases—posterior tilting of the EV-ICD lead. Specifically, this phenomenon refers to the distal portion of the lead shifting its position toward the posterior mediastinum while the proximal portion remains secured anteriorly, near the sternum.

## Case reports

### Case 1

A 36-year-old woman with a history of obesity (body mass index [calculated as weight in kilograms divided by height in meters squared] = 27.8), hypothyroidism, and psoriasis presented with ventricular fibrillation and was successfully resuscitated. Subsequent electrocardiography findings (sinus rhythm 75 beats/min, PR interval 132 ms, QRS duration 108 ms, corrected QT interval 445 ms, and no signs of ischemia) were normal, as was coronary angiography. Echocardiography and cardiovascular magnetic resonance (CMR) studies revealed a dilated left ventricle and severely reduced left ventricular ejection fraction of 13%. Intensive transmural late gadolinium enhancement was visible. Endomyocardial biopsies from both the right and left ventricles, along with whole-body positron emission tomography-computed tomography (PET-CT), ruled out an inflammatory cardiomyopathy. Genetic analysis failed to identify any pathogenic variants.

The diagnosis of dilated cardiomyopathy was established without a specific etiology. The patient experienced a favorable neurologic recovery. Considering the patient’s young age, the associated risk of future lead failure, and the potential need for transvenous lead extraction, along with the absence of a requirement for cardiac pacing, the EV-ICD was chosen and implanted 25 days post event. The pre-implantation CT and CMR scans showed a large substernal space. The implantation was uneventful with a single tunneling, no tip movement of the lead, and good electrical values (R-wave sensing 2–3 mV). Defibrillation threshold (DFT) testing was successful with 30 J.

**Figure 2:**
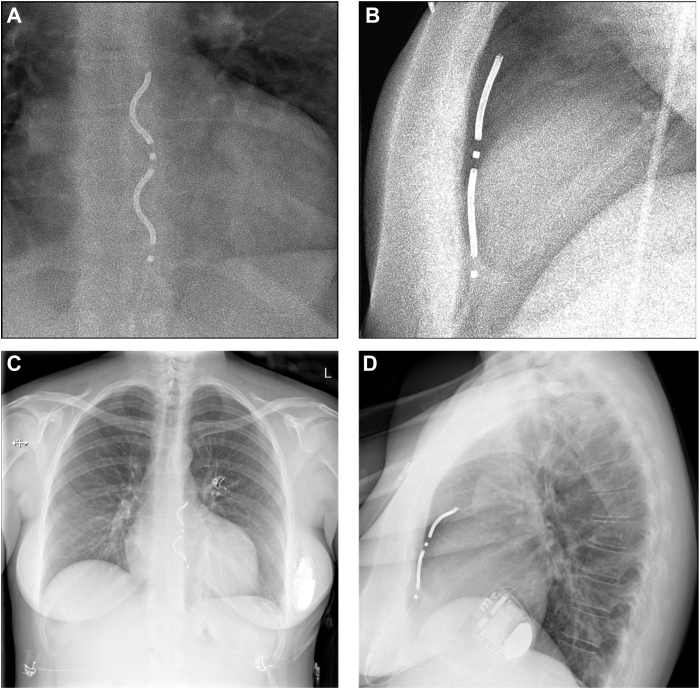
Extravascular implantable cardioverter-defibrillator (EV-ICD) lead position in case 1. EV-ICD lead position during the implantation from the anteroposterior (**A**) and the left anterior oblique 90-degrees (**B**) views. Post-implantation chest radiograph shows unchangeable lead position in the anteroposterior view (**C**), but posterior tilting in the lateral view (**D**).

The final fluoroscopy image depicted the lead in an optimal position beneath the sternum. However, chest radiography on the subsequent day revealed a tilt of the lead tip posteriorly toward the heart (Figure 2). The patient experienced left-sided chest wall discomfort, exacerbated by deep breathing, necessitating analgesic treatment with paracetamol and tramadol. The electrical performance of the lead remained stable; the R-wave sensing improved up to 4 mV and no atrial oversensing was detected in the electrogram. The chest pain subsided rapidly, enabling the patient's discharge 2 days post implantation.

**Figure 3:**
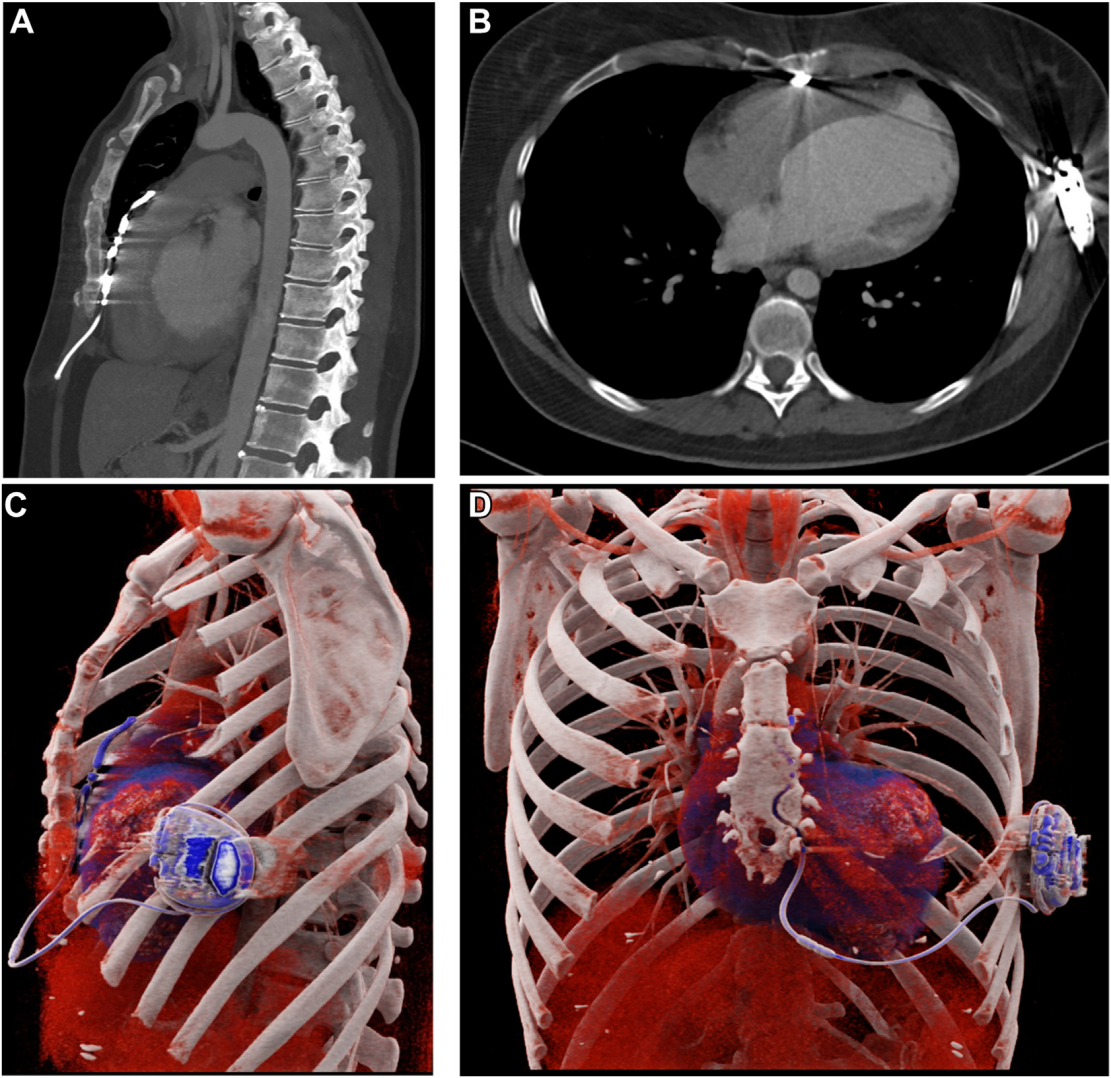
Postoperative computed tomography (CT) of case 1. The CT images (**A, B**) and 3-dimensional (3D) reconstruction (**C, D**) show posterior tilting of the extravascular implantable cardioverter-defibrillator lead toward the heart. The ribs were partly removed from the 3D reconstruction to visualize the position of the device. Image **B** shows the relatively narrow space between the inferior part of sternum and the cardiac structures can be seen. The retrosternal space is larger at the level of middle and superior one-third of the sternum. Images **A** and **B** show the streak-like, beam-hardening artifacts induced by the metallic pacing lead.

To verify the position of the EV-ICD lead tip, an elective chest CT scan with intravenous iodine contrast agent was obtained. The EV-ICD lead was located in the anterior mediastinum, resting on anterior paracardial fat. The pericardium could not be distinguished as a separate structure throughout the entire course of the lead, but no pericardial effusion or abnormal pericardial contrast enhancement was noted (Figure 3 and Supplemental Video). The position of the lead was interpreted to be appropriate, indicating no need for corrective measures.

At 1-month and 6-month follow-up visits, the patient was asymptomatic and had no arrhythmic events or oversensing episodes. Furthermore, the lead impedance had remained stable, and R-wave sensing was consistently good, ranging from 3.5 to 5 mV during the in-office testing. There have been no alerts in the remote follow-up of the device.

### Case 2

A 43-year-old woman (body mass index = 19.6) was referred for further evaluation of mitral valve prolapse syndrome. On transthoracic echocardiography, both mitral valve leaflets were thickened and prolapsing. Mitral regurgitation was determined to be mild, and the function of both ventricles was normal. A prominent mitral annular disjunction (10 mm) was noted, and the patient also exhibited T-wave inversions in the inferior leads on a 12-lead electrocardiogram, suggestive of arrhythmic mitral valve prolapse syndrome. CMR further revealed an area of late gadolinium enhancement in the inferolateral peri-annular region of the mitral valve apparatus. Holter monitoring documented episodes of nonsustained ventricular tachycardia and a moderate burden (5%) of polymorphic premature ventricular contractions. The patient reported long-standing palpitations, but she had no history of syncope, heart failure symptoms, or exercise limitations. According to the European Society of Cardiology consensus document,^4^ the patient’s findings supported consideration of ICD implantation. After a thorough discussion, the patient opted for the procedure.

Given her young age and the absence of need for antibradycardia pacing, the advantages and disadvantages of nontransvenous device options were discussed. The patient chose the EV-ICD. A preprocedural chest CT scan confirmed that there was a normal space between the posterior sternum and anterior cardiac structures.

The lead was placed under the left-sided part of the sternum without any resistance during the tunneling. The first lead position was accepted with an excellent ventricular sensing of 5.7 mV without discriminable P wave. DFT testing was successful with 30 J.

**Figure 4:**
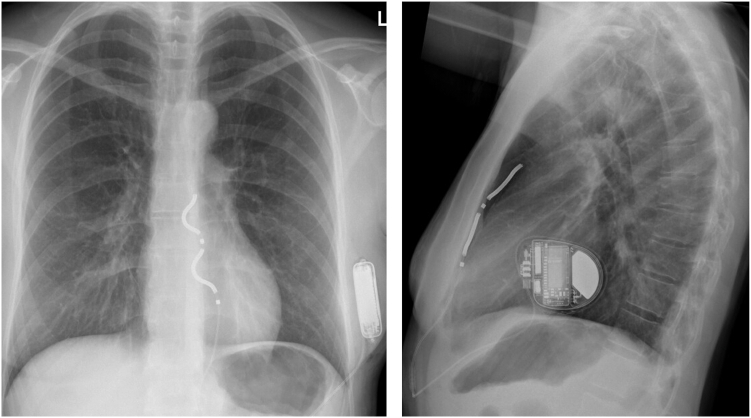
Postoperative chest radiograph of case 2. After an uneventful implantation, the extravascular implantable cardioverter-defibrillator lead demonstrates a tilt toward the heart.

All antitachycardia therapies remained deactivated until the next day to ensure stable lead positioning and appropriate sensing. The patient reported feeling well post procedure, with only mild chest discomfort. However, a chest radiograph obtained 5–6 hours after implantation showed that the distal coil of the substernal ICD lead had tilted toward the heart (Figure 4). The electrical performance of the lead remained stable without a significant change in the ventricular sensing: R-wave measurements were 4.4 mV in the supine position, 2.9 mV when lying on the right side, 3.2 mV on the left side, and 2.2 mV while standing.

The lead tip stayed stable at this position the day after, and a CT scan confirmed that no cardiac or pleural structures were injured by the lead. Over the 6-month follow-up period, all electrical parameters remained stable with R-wave sensing of 3.9 mV in the supine position during the latest in-office evaluation.

### Case 3

A 38-year-old obese (body mass index = 28) man with a history of dilated cardiomyopathy was referred for evaluation of primary prevention ICD. Eight months earlier, he had presented with New York Heart Association class IV heart failure symptoms, sinus rhythm with narrow QRS complexes, and a severely reduced left ventricular ejection fraction of 10%. After 6 months of contemporary heart failure management, the patient showed significant clinical improvement, presenting with New York Heart Association class I heart failure. Genetic testing was inconclusive. CMR demonstrated slightly improved yet severely depressed left ventricular ejection fraction (20%). A thorough discussion on primary prevention ICD implantation was held, including the various types of devices. Given the patient’s young age and no need for pacing, a transvenous device was not considered as the first choice. Instead, considering the greater battery longevity compared with a subcutaneous ICD and the option for antitachycardia pacing, the patient opted for EV-ICD. CMR confirmed that the distance between the posterior sternum and anterior cardiac structures was suitable for the procedure.

The initial lead position was accepted with ventricular sensing of 4.5 mV. DFT testing was successful with a 30-J shock. Following institutional protocol, antitachycardia therapies were deactivated until the first device interrogation the following day. A chest radiograph obtained 6 hours post implantation showed the lead and device in the correct position with no evidence of pneumothorax. Despite paracetamol, the patient reported continuous retrosternal and right-sided nonrespiratory pain, which was managed by adding tramadol to his analgesic regimen.

**Figure 5:**
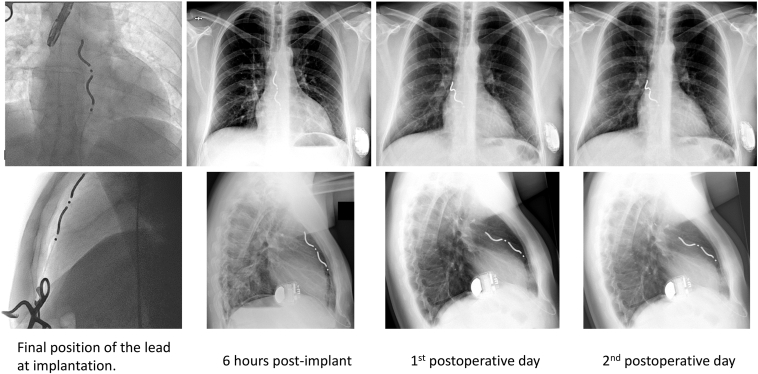
Extravascular implantable cardioverter-defibrillator lead position change in case 3. After the initial substernal position, the lead has tilted posteriorly and shifted slightly to the right during the 2 days to follow-up.

At the next day's device follow-up, the R-wave sensing had decreased to 2.2–2.8 mV, with no discernible P wave in any position (eg, supine, right/left lateral, or sitting). Despite the decline, sensing remained within the normal range for the EV-ICD, with no oversensing detected and stable high-voltage impedance. Considering the balance of risks and benefits, DFT retesting was deemed unnecessary. The patient continued to report right-sided nonrespiratory pain, prompting follow-up radiography. The image revealed that the lead had tilted backward toward the heart silhouette and shifted slightly to the right. The position of the proximal electrode, however, was unchanged, indicating that the fixation of the lead was uncompromised (Figure 5).

By the third post-implantation day, the pain was controlled with paracetamol alone. At discharge on day 4, device follow-up showed stable impedance and sensing values. Telemonitoring was initiated and the therapies were activated. The 3-month follow-up was uneventful, with normal device function and R-wave sensing of 2.7 mV during the latest in-office evaluation.

## Discussion

We reported on 3 patient cases in which posterior tilting of the substernal EV-ICD lead was observed after implantation. In all of these cases, the lead was initially implanted close to the posterior surface of the sternum, and the tilting of the lead toward the heart occurred shortly afterward (within 24 hours). Despite the tilting of the lead, the EV-ICDs remained fully functional and no corrective procedures were necessary. Although the relatively stable electrical parameters of the tilted leads are reassuring, we cannot be certain about the DFT in the new lead position. However, given the faultless sensing and the closer proximity of the lead to the heart, retesting was not deemed to offer sufficient benefits to outweigh the potential risks^5^ in these cases. Two of the 3 patients reported chest discomfort requiring analgesia. This can be speculated to have occurred due to the parietal pericardial leaflet irritation. The sensations resolved promptly.

Assessing lead position on chest radiographs relies on anatomic landmarks. Comparison with the previous imaging studies and the fluoroscopic images taken during implantation is crucial for accurate evaluation. Although chest radiography is the first-line imaging modality for assessing lead position, CT imaging offers more precise anatomic localization of pacing leads and allows for the creation of 3-dimensional reformats. However, metallic leads induce artifacts on CT images, occasionally complicating interpretation. If the lead position remains unclear on chest radiograph, or if complications are suspected, CT is the imaging modality of choice.

The pericardium is a thin anatomic structure with 2 leaflets that create a potential space, typically containing 15–50 mL of serous fluid. On CT images, >4-mm thickness is considered abnormal, although fluid-filled pericardial recesses may appear thicker. The pericardium is not always clearly visible on CT at all sites. Nevertheless, irritation or inflammation of the pericardium usually results in clearly visible findings on CT images, such as thickening and contrast enhancement of the pericardial leaflets and pericardial effusion.^6^

In patient case 1, the pericardium is clearly visible adjacent to the EV-ICD lead at the site, where the lead enters substernal space. Between the sternum and right ventricle free wall, the substernal space is narrow, and the artifacts obscure visualization of the pericardium. No signs of pericardial irritation were noted on CT in cases 1 and 2. The anterior mediastinal fat is continuous with the paracardial fat retrosternally. We speculate that dislodgement of a lead tip situated in the substernal space may occur due to the movement of the thoracic wall and mediastinal structures during respiration and cardiac motion.

CT scans were used in cases 1 and 2 to verify the lead position following the exceptional lead position in the chest radiograph. Also, in case 3, a CT was planned but was refused by the patient. Based on our experience with these cases, we believe that a CT scan may not be necessary for future patients if lead tilting occurs after conventional tunneling, provided that the postoperative echocardiogram shows no signs of pericardial effusion, and the electrical parameters remain within acceptable ranges. However, during the implantation it remains important to monitor the lead tip movement and the lead position in both anteroposterior and lateral views to avoid pericardial or pleural implantation. Initial experience from EV-ICD lead extraction has shown high success.^7^ The posterior tilt of the lead may make the removal of the lead more difficult, especially if dedicated extraction tools are needed instead of simple traction.

## Conclusion

Following successful implantation of an EV-ICD, tilting of the ICD lead can occur, particularly if the space between the sternum and the heart is relatively large. Based on the cases we presented, we believe that posterior tilting of the EV-ICD lead is a benign phenomenon and does not compromise device function. Our experience may help other centers with similar cases.

## Declaration of Generative AI and AI-Assisted Technologies in the Writing Process

During the preparation of this work the authors used ChatGPT 4.0 in order to improve readability and language. After using this tool, the authors reviewed and edited the content as needed and take full responsibility for the content of the publication.

## Disclosures

Jarkko Karvonen: Speaker honoraria and consultancy fees from Abbott, Biotronik, Boston Scientific and Medtronic, and Medtronic Micra Advisory Board.

Aino-Maija Vuorinen: Speaker fees from The Radiological Society of Finland and Finnish Cardiac Society; stock: Osgenic Oy. Fabian Noti: Medtronic Advisory Board EV-ICD; travel fees/educational grants Medtronic, Biotronik, Boston Scientific, Abbott; all without personal remuneration. Andreas Haeberlin: Dr. Haeberlin serves as a proctor and consultant for Medtronic. He has received travel/educational/research grants from Medtronic, Biotronik, Abbott, Boston Scientific and Philips/Spectranetics without impact on his personal remuneration. He has received research grants from the Swiss National Science Foundation, the Swiss Innovation agency Innosuisse, the Swiss Heart Foundation, the University of Bern, the University Hospital Bern, the Velux Foundation, the Hasler Foundation, the Swiss Heart Rhythm Foundation, and the Novartis Research Foundation; he is co-founder and clinical head of Act-Inno AG. Alexander Breitenstein: Consulting and speaker fees from Abbott, Bayer Health Care, Biosense Webster, Biotronik, BMS/Pfizer, Boston Scientific, Cook Medical, Daiichi Sankyo, Medtronic, Philips, and Zoll.
