# Supplementary figures and images for: Unexpected posterior tilt in extravascular implantable cardioverter-defibrillator leads: Lessons learned from 3 cases
Source: HeartRhythm Case Rep. 2025 Jan 30;11(4):347–53. doi: 10.1016/j.hrcr.2025.01.010 (PMC12138115; doi:10.1016/j.hrcr.2025.01.010)

## Slide 1
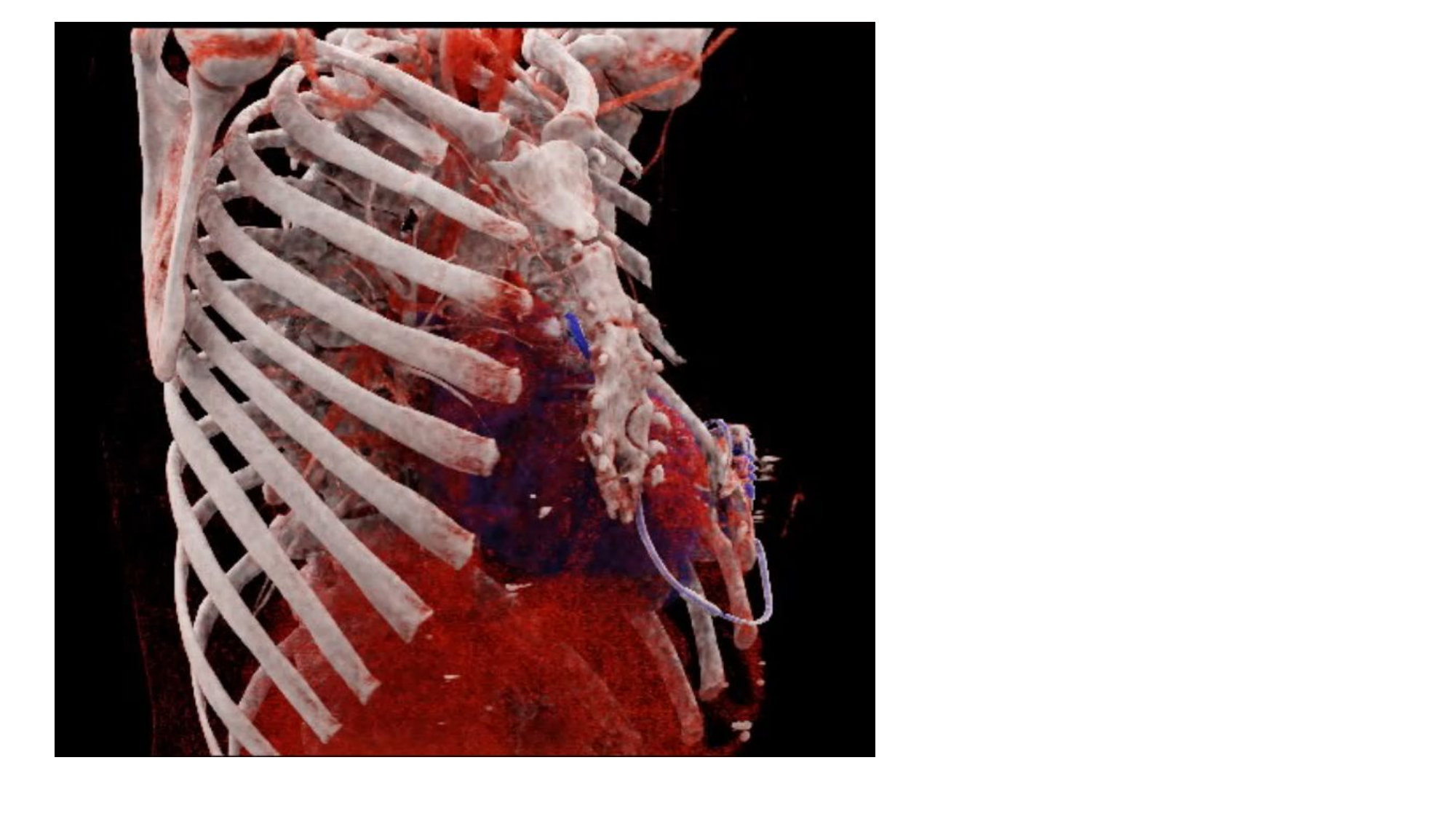

Supplement: Supplementary Video 1 [file mmc1.pptx]
