# Supplementary material for: Unexpected posterior tilt in extravascular implantable cardioverter-defibrillator leads: Lessons learned from 3 cases
Source: HeartRhythm Case Rep. 2025 Jan 30;11(4):347–53. doi: 10.1016/j.hrcr.2025.01.010 (PMC12138115; doi:10.1016/j.hrcr.2025.01.010)
Supplement: Supplementary Video 2 [file mmc2.docx]

**Supplementary video legend**

**Postoperative CT of Patient #1.** 3D reconstruction of the post-implantation CT shows the posterior tilting of EV-ICD lead.
